# A Tough Bullet to Swallow

**DOI:** 10.14309/crj.0000000000001887

**Published:** 2025-11-17

**Authors:** John A. Cooper, Elizabeth Statham, Stephen Brown, Shajan Peter

**Affiliations:** 1 Department of Internal Medicine, University of Alabama at Birmingham, Birmingham, AL

## CASE REPORT

Gunshot wounds to the neck may result in complex injuries, including rare instances of ballistic fragment ingestion or migration.

**Figure 1.**
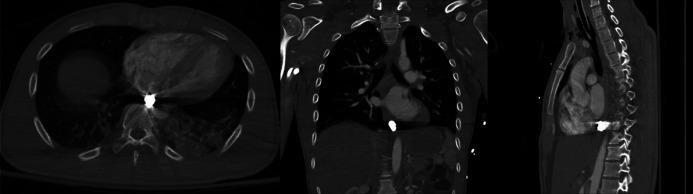
Computed tomography imaging of swallowed bullet.

**Figure 2.**
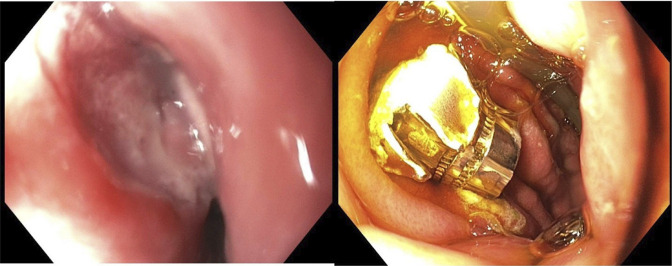
Endoscopic image of swallowed bullet.

A 27-year-old man presented after a Gunshot wound to the posterior pharyngeal region. Computed tomography angiogram of the chest and abdomen revealed a ballistic fragment in the posterior mediastinum near the diaphragm, within the expected location of the esophageal lumen (Figure 1). No soft tissue gas, fracture, or vascular injury was present, suggesting esophageal entry rather than direct mediastinal trauma. The patient developed hemiparesis, necessitating MRI, which was delayed due to the metallic fragment. Gastroenterology was consulted for endoscopic removal. Esophagogastroduodenoscopy, performed approximately 36 hours after injury, revealed ulceration in the subglottic space, likely representing the bullet's entry site (Figure 2). The fragment was located distal to the gastroesophageal junction and retrieved using a Roth net (Figure 2).

Early endoscopy in penetrating pharyngeal trauma remains controversial but can be safely performed in stable patients without perforation. In this case, it enabled successful diagnosis and retrieval without complications. Clinicians should consider endoscopy in stable patients with suspected ingestion or migration of ballistic fragments, especially when MRI is indicated and no perforation is evident.

## DISCLOSURES

Author contributions: J. Cooper: Manuscript compilation and review; E. Statham, S. Brown, and S. Peter: Manuscript review. S Peter is the article guarantor.

Financial disclosure: None to report.

Informed consent was obtained for this case report.

## ABBREVIATIONS:

MRI: magnetic resonance imaging

